# Revisiting the public–private wage gap in Spain: new evidence and interpretation

**DOI:** 10.1007/s13209-023-00277-z

**Published:** 2023-04-25

**Authors:** Alba Couceiro de León, Juan J. Dolado

**Affiliations:** Department of Economics UC3M, Universidad Carlos III de Madrid, c/Madrid 126, 28903 Getafe, Madrid, Spain

**Keywords:** Public sector, Private sector, Public–private wage gap, Monopsony, Unions, J31, J38, J42, J45

## Abstract

This paper updates the available evidence on the public–private wage gap in Spain, which dates back to 2012. Through microdata drawn from the last three waves of the Wage Structure Survey (2010, 2014 and 2018), we study how this gap and its distribution by gender and education have evolved during and after the Great Recession. Conventional Oaxaca-Blinder decompositions are used to divide the raw wage gap into a component explained by differences in characteristics and another one capturing differences in returns and endogenous selection. The main findings are: (i) a strong wage compression by skills, and (ii) a wage premium for less-skilled women in the public sector. Both empirical results can be rationalised by a monopoly union wage-setting model with monopsonistic features and the presence of female statistical discrimination.

## Introduction

The public sector plays a very influential role in labour markets with its employees representing between 15 and 20% of total salaried employment in many advanced economies. In particular, jobs in the public and private sectors differ in both the nature of the productive tasks they perform (since the public sector is the only provider of certain goods and services except in education and health) and in their working conditions (see, e.g. Garibaldi and Gomes 2020). Accordingly, not only the public sector may enjoy some monopolistic rents but it also becomes a bilateral monopsony in a segment of the labour market that is marked by a strong union establishment and has its own wage-setting mechanisms and recruitment selection procedures.

As a result of these features, public sector employees exhibit specific characteristics—particularly in relation to gender, age or educational attainments—which differ from those present in the private sector. These differences translate into wage gaps which experience substantial variation across workers and also lead to disparate wage distributions in each sector. For example, a stylized fact in most developed countries is the greater compression of public sector wages across different levels of education: less-skilled public employees' pay is higher than in the private sector, while the opposite holds among high-skilled employees.

Spain is no exception to these empirical regularities, as evidenced in an emerging literature on the public–private wage gap that began in the late 80 s but has become somewhat outdated.^1^ Indeed, to our knowledge, the latest available research on this wage gap (see Hospido and Moral-Benito 2016) dates back to the years 2005–2012, that is, a period preceding the end of the sovereign debt crisis and the subsequent recovery of the Spanish economy after 2014. To help fill this gap, our goal in this paper is to update the evidence to 2018.^2^ In effect, while private employment soared during the early 2000s and then plummeted since 2008, public employment and wages experienced large swings, growing until 2011 to then decline until 2013. These fluctuations resulted from the initial stimulus plans (up to 2011) implemented by the Spanish government to fight the impact effects of the Great Recession and the subsequent fiscal consolidation plans (up to 2013) agreed with European authorities (see, e.g. Christofides and Michael 2020). However, once the recovery phase started in 2014, employment in both sectors picked up though at a faster rate in the private sector. Hence, revisiting the empirical evidence on the public–private wage differential beyond the early 2010s helps get a broader picture of how it has evolved in circumstances quite different from those prevailing in the existing literature.

Before summarising our main results, it is worth discussing the choice of the data source that will be used in this paper. To overcome the limitations arising from the scarcity of high-quality data on labour earnings and the use of relatively small samples in the early studies of the 80 s and 90 s, two major advances in data collection have brought substantial research progress with the turn of the century. On the one hand, there is the availability of rich longitudinal information since 2005 in the *Continuous Sample of Working Histories* (Muestra Contínua de Vidas Laborales–MCVL in short). By linking a large sample of administrative records of the social security to income tax data, the MCVL provides information on annual wages in both sectors, together with several basic job characteristics for samples exceeding 1 million individuals each year. This database, used by Hospido and Moral-Benito (2016) among others, includes information regarding those public sector employees who are affiliated to the General Regime of the Spanish pension system. On the other hand, there have been substantial data-collection improvements in the new waves of the *Wage Structure Survey* (Encuesta de Estructura Salarial—EES) which, by being harmonised with other similar wage surveys in Europe, has traditionally been considered the most detailed source of information on labour earnings in Spain. Carried out on a four-year basis through a two-stage stratified sampling design at the establishment-worker levels, it collects information on monthly wages and hours of work of employees in the private and public sectors, with samples sizes around 200,000 per wave. The latter are civil servants (*personal funcionarial*) and other types of employees (*personal laboral*) in Industry, Construction and Services who, as in the MCVL, are affiliated to the General Regime of the Social Security system.

When comparing both data sources, several pros and cons emerge. The main advantage of EES is that it provides more accurate information regarding employees' education and occupation, whose codifications in MCVL are fairly outdated. Moreover, unlike the MCVL, the EES includes data for the two regions with specific tax regulations (Navarra and País Vasco). Just as relevant is the fact that the latter collects detailed information on type of collective bargaining and hours of work—which are not recorded in MCVL.^3^ Accordingly, we use the three more recent waves of the quadrennial EES (2010, 2014 and 2018) as our database in the rest of the paper, though this choice is not free of problems. In effect, the main disadvantage of using EES is that, as with the MCVL, information on household and family background is very scarce. Hence, by only focusing on employees, it becomes difficult to account for selection into either sector, implying that results will only be representative for individuals in work. However, the EES provides information on maternity and paternity leaves that will allow us to somewhat identify selection corrections for each sector and by gender. Admittedly, this problem can be alleviated by using the MCVL since its longitudinal design allows to control for individual effects affecting sorting and wages in standard or quantile *mincerian* panel regression models. In addition, it has been argued that survey data could be a matter of concern, again due to non-classical measurement error, though the two-stage stratified design of the EES is likely to reduce this problem (see Casado and Simón 2015). At any rate, we think that, according to the reasons explained above, the EES database used in the sequel incorporates some advantages over previous attempts to measure public–private hourly wage gaps in Spain.

Summing up, the questions we try to answer in the sequel are basically whether the recovery phase since 2014 and the effects of the labour market reforms implemented in 2010 and 2012 have brought relevant changes in the regularities uncovered during the Great Recession. Our main findings can be summarised as follows. We document: (i) a wage gap of about 6 points on average in favour of the public sector which is not explained by differences in productivity, (ii) wage compression, with a positive (resp. negative) gap for public employees with less (resp. higher) qualifications and (iii) a wage premium for less-skilled women working in the public sector. In general, these results are qualitatively similar to those reported in the literature but there is some divergence in quantitative terms on which we try to shed light through some theoretical interpretations.

The rest of the paper is organised as follows. Section 2 reviews the existing literature on the differences in employment and wages between both sectors. Section 3 summarises the main descriptive statistics of the EES dataset used in the empirical sections. Section 4 presents the empirical strategy. Section 5 reports the main results. Sections 6 suggests a rationalisation of the main findings. Finally, Sect. 6 concludes. An Appendix gathers several additional Tables and results.

## Related literature

Public and private sector wages may diverge for multiple reasons, among which differences in workers' socioeconomic characteristics, non-competitive wage setting, employers' objectives and union strength stand out. In effect, factors such as education, experience, gender, age or wage-bargaining regulations differ between the two sectors, thus generating a high degree of heterogeneity in their wage gap.

As already pointed out, a stylized fact that has been studied extensively in the literature is the compression of public wages along the wage and the skill/qualification distributions. As regards jobs held by less-skilled individuals, the public–private wage gap is found to be positive in most countries. On the contrary, a negative gap is observed among workers with higher education. In early contributions to this topic, Borjas (1980, 1984) found a growing compression of public wages in the US from the 1970s onwards, as well as a persistent change in worker flows between sectors. Both findings would explain the increasing difficulty of the US public sector to attract and retain most-qualified workers. As regards the European Union (EU) member states, Campos and Centeno (2012) also conclude that the public–private wage gap narrows along the distribution of skills before the adoption of the euro. Using a fixed-effects approach with longitudinal data from the *European Community Household Panel* (ECHP) survey, they claim that the public sector attracts the best-qualified individuals for jobs at the bottom of the wage distribution (over-education), but fails to retain the most skilled workers in the best-paying jobs. Along the same lines, Giordano et al. (2011) use the *European Union Statistics on Income and Living Conditions* (EU-SILC) microdata to argue that the public–private wage gap is greater in Spain, Italy, Greece, Portugal or Ireland than in central European countries. Likewise, the standard Oaxaca-Blinder (OB) decompositions used by Castro et al. (2013), Christofides and Michael (2013) and Depalo et al. (2015), among many others, evidence that the unexplained part of the public–private wage gap is negative in some Scandinavian countries while it is positive in southern Europe.

Other salient features reported at length in the previous literature are the high representation of high-educated workers and women in the public sector. For example, regarding the first fact, Garibaldi et al. (2021) argue that public employment is skewed towards high-skilled labour because governments seek to get better inputs for the production of public goods and services, as well as hire highly qualified staff since they are relatively less costly when public wages are compressed along the skills distribution. As for the second fact, Garibaldi and Gomes (2020) document that the fraction of women in the public sector of 20 OECD countries greatly exceeds their corresponding share in total employment. Yet, only a few studies try to rationalise this fact. Among them, De la Rica et al. (2007) argue that unemployed or inactive women are much more likely to seek public sector jobs than men to avoid statistical discrimination in the private sector, due to their greater job instability, especially when they have small children or are in fertility age. Similarly, by means of a calibrated search and matching model, Gomes and Kuehn (2019) claim that female overrepresentation in the public sector is not due to labour demand forces but rather to a greater supply of women who have stronger preferences for working in the public sector than men. Thus, these studies conclude that women value more the compensation offered by the public sector in the form of reconciling personal and professional lives, in addition to providing more protection against some potential discrimination.

Note that the existence of a positive public–private wage gap goes against the theory of compensating differentials, which predicts that, *ceteris paribus*, jobs with higher risk or fewer comforts should be compensated with higher pay. The available evidence has shown that public jobs are more stable than jobs in the private sector, where the risk of dismissal is higher. Chassamboulli et al (2020) and Fontaine et al. (2020) analyse labour market flows between the public and private sectors in France, Spain, the UK and the USA, finding lower turnover and job finding rates in the public sector and a higher separation rate in the private sector. Based on similar findings, Gomes (2016, 2018) suggests that, due to greater job security in the public sector and its lower rate of job destruction, the wage differential should be approximately 2.5% higher in the private sector.

In addition, there is a line of research on the role that public wages play as a decision variable to maximise political support. For example, Alesina et al. (1999) argue that governments may ignore efficiency criteria for determining public wages and employment and instead often choose to divert them towards influential minorities and political groups.

Finally, regarding the specific literature focusing on the public–private wage gap in Spain, there are several studies related to ours. For example, among the more recent ones, García-Pérez and Jimeno (2007) use the panel dimension of the ECHP survey from 1995 to 2001 to estimate characteristics-adjusted wage gaps across Spanish regions. They find that these gaps are mostly explained by differences in returns rather than by differences in characteristics or selection effects, and that they are higher in less productive regions. However, their samples are very small and the definition of public employees in the ECHP in less accurate than in other data sources, like EES or MCVL. Similar shortcomings affect the EU-SILC dataset used by Giordano et al. (2011) who find a slightly larger unexplained component of the gap among women than among men. Undoubtedly, the closest paper to ours is the above-mentioned study by Hospido and Moral-Benito (2016). They use richer longitudinal data from the MCVL (2005–2012) to show that: (i) a raw gap of 35 percentage points (pp.) in favour of public sector employees is reduced to 10 pp. once observed characteristics and endogenous selection are accounted for, (ii) the public wage premium is highly procyclical reaching a peak in 2009 and declining afterwards and (iii) workers at the top of the wage distribution select negatively into the public sector while the opposite holds at the bottom of the wage distribution. Our results drawn from the EES differ from theirs in that selection seems to be less important, except for less-educated women, possibly due to the richer set of controls available in that dataset. Moreover, the magnitude of the raw public (hourly) wage premium is lower than the one they report, perhaps reflecting measurement errors from their working hours imputation. Yet, in line with their results, we find that the wage premium is procyclical, increasing after the beginning of the recovery period around 2014. Lastly, we provide some theoretical underpinnings for our findings in terms of three important factors: (i) the bilateral monopsonistic position of the public sector, (ii) the objectives of public sector unions and (iii) the potential statistical discrimination effects suffered by less-educated women in the private sector.

## Data

As discussed above, our data source is the quadrennial EES survey that we believe provides more reliable information than other alternative databases in terms of wages, hours of work and a wide range of demographics and firms' characteristics. We use data on public and private employees from the three last available waves of the EES (2010, 2014 and 2018) where military personnel and individuals under 19 years of age and over 59 have been excluded to homogenise the sample in both sectors. In particular, the 2010 wave allows us to compare our findings with those reported by Hospido and Moral-Benito (2016) for the MCVL (2005–2012) given that this year overlaps in both samples. Descriptive statistics for both sectors are presented in Table 8 (public sector) of the Appendix.

In line with other countries, the first relevant feature is the overrepresentation of women in the Spanish public sector: they account on average for around 55% of employees against 41% in the private sector. Another salient difference is the greater fraction of high-skilled individuals in the public sector (30% have a college or even higher degree vs. 17% in the private sector), which also translates into a higher weight of upper occupations (A0 to D0): 42% vs. 32%.

Further differential patterns are related to age and job tenure: on average 34% of public employees are above 50 years of age vs. 20% in the private sector, while tenure is about 5 years longer. Both figures point to a much higher job stability in the public sector which may be related to the greater proportion of women enjoying maternity leaves in that sector (despite being older on average). As regards labour contract types, temporary contracts are higher in the public sector (31.7% vs. 18.6%) while the opposite holds for part-time work (9.0% vs. 17%).^4^ In any case, outflows to unemployment and inactivity are much lower in the public than in the private sector (see Fontaine et al. 2020).

**Fig. 1:**
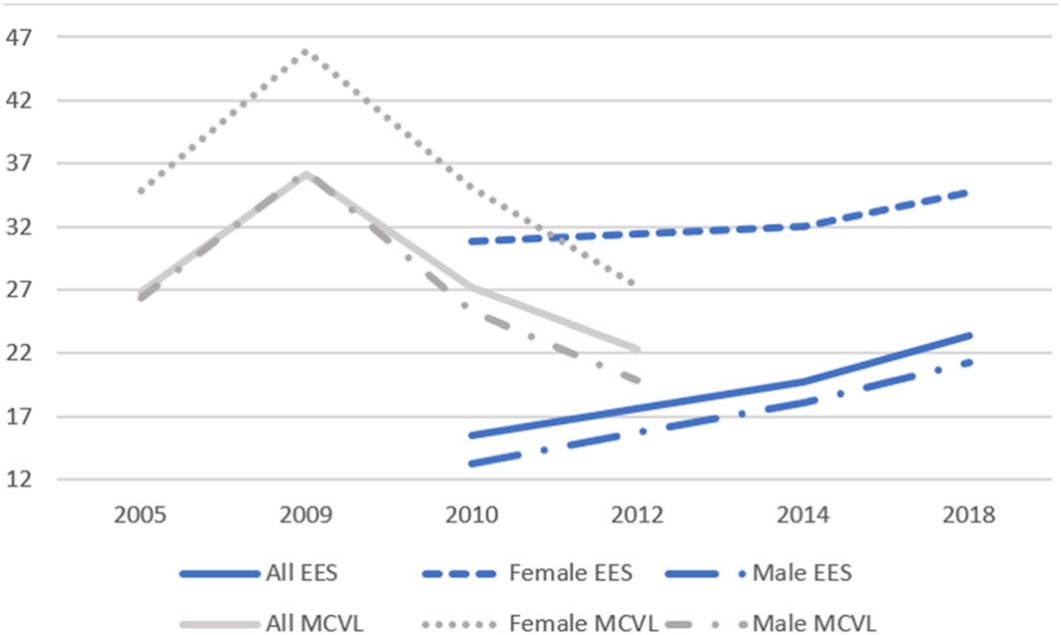
Raw public–private wage gaps: EES vs. MCVL (whole sample and by gender)

Figure 1 presents the raw public–private wage gap measured in per cent of private sector wages for all, female and male individuals in each of the EES waves. For comparison, the corresponding raw gaps reported by Hospido and Moral-Benito (2016) for the selected years 2005, 2009, 2010 and 2012 are also included. As can be seen from their figures, the public–private wage gap increases from 2005 to 2009, reaching a peak of 36% in that year, to then fall to 22% by 2012. Our figures in turn show a steady rise from 2010 until 2018, confirming the procyclicality of the wage premium. Interestingly, the overall raw gap with the MCVL is 11.7 pp. higher (= 27.2–15.5) in the common year of both samples, possibly due to measurement errors in the MCVL since working hours are imputed from other databases. At any rate, the results by gender agree in both studies: women in the public sector enjoy much higher wage premia than men.

## Empirical strategy

The dependent variable in all subsequent regressions is the (logged) hourly wage of individual $i$, $\mathrm{ln}\left({w}_{i}\right)$, computed as the ratio between the monthly wage (defined as the sum the base salary, monthly extraordinary pay, overtime payments and salary supplements) and monthly working hours (defined as the sum of agreed and overtime weekly working hours). Both wages and hours are collected in October of each year, since this represents a “normal” month as regards seasonal payments or holidays.

Following the literature on the public–private wage gap, we first estimate a *mincerian* earnings equation by pooled OLS, where $\mathrm{ln}\left({w}_{i}\right)$ is regressed on an indicator variable for public sector employment, ${PUS}_{i}$, and a vector of sociodemographic controls, ${{\varvec{X}}}_{i}$ (age, gender, education, etc.) To capture the gender-differential effect of working in the public sector, an interaction term between ${PUS}_{i}$ and a female indicator, ${Fem}_{i}$, is also included, leading to the following regression: 1 where ${\varepsilon }_{i}$ is a zero-mean, uncorrelated and homoscedastic error term. As regards the set of observable controls, ${{\varvec{X}}}_{i} ,$ two specifications (I and II) are considered. The first one is a standard *mincerian* wage equation including dummy variables for human capital and location, such as age (3), region of residence (6), and education (7), plus tenure and tenure-squared. The second one extends the previous set of controls by adding labour demand covariates, such as dummies for fixed-term contract (1), part-time work schedule (1), type of collective bargaining (3), occupation (15) according to the categories established in the National Classification of Occupations CNO-11.^5^ Concerning the different types of collective bargaining, the EES distinguishes among sectoral/state or lower levels (regional, provincial, etc.), firm and work centre, and other forms of regulations which include specific wage agreements for civil servants. To select the maintained specification of the set of controls in the rest of the paper, both models are estimated for the whole sample of individuals in each of the three waves.^6^

Pooled OLS regressions implicitly impose the same returns to observed characteristics for the two sectors, so that the public–private wage gap only depends on the ${PUS}_{i}$ shift factor and its interaction with gender. A conventional method in the literature to relax this restriction is the use of the Oaxaca-Blinder (OB) decomposition, once wage regressions are run separately for each sector: $\mathrm{ln}\left({w}_{Si}\right)={\beta }_{S}{X}_{Si}+{\varepsilon }_{Si} ,$ with $S=${$G$=public, $P$=private}. In this fashion, the gap in mean logged wages, $\overline{\mathrm{ln }({w}_{G})}- \overline{\mathrm{ln }\left({w}_{P}\right),}$ is split into two components: an *explained* part justified by disparity in observed characteristics (for given returns), $\left(\overline{{x }_{G}}-\overline{{x }_{P}}\right)\widehat{{\beta }_{G},}$ and an *unexplained* one stemming from differences in returns (for given characteristics), $\overline{{x }_{P}}(\widehat{{\beta }_{G}}-\widehat{{\beta }_{P})}$.

A common concern with this approach is that, by ignoring the endogeneity of sectoral choice in the OLS regressions, estimated returns could be biased. Lacking longitudinal data to control for fixed effects, we address this issue with a conventional switching regression (SR) approach. The SR model includes two wage equations (one for each sector) and the selection equation, assuming joint Gaussian dependence of the error terms in each wage and selection equations. This leads to a standard two-step Heckit estimation approach where the inverse Mill's ratio of the public sector choice, is estimated with a *probit* model (${PUS}_{i}$ =1), and then is added to the set of covariates in the wage equations. To address the identification problem—besides age, location and education—we include in the probit some variable that is likely to influence the choice of the sector but not the wage. In particular, lacking other household characteristics in the EES, we use the information on maternity and paternity leaves as proxies for the presence of small children. As usually argued, these can motivate workers' preferences for more stable jobs and non-pecuniary benefits like those typically linked with public employment. Accordingly, the unexplained part in the OB decomposition gets augmented by the difference in selectivity terms, ${\theta }_{G}{\lambda }_{G}-{\theta }_{P}{\lambda }_{P}$, where ${\lambda }_{S}$ ($S=G,P$) are the scaled inverse Mills' ratios, and the statistical significance and the sign of the ${\theta }_{s}$ coefficients indicate whether selection is relevant and if it is positive or negative (Tables 1, 2, 3, 4 and 5).

**Table 1:** OLS estimates in wage regressions (whole sample) (a) Specification I (b) Specification II

	2010	2014	2018	2010	2014	2018
PUS	0.053 (0.003)	0.025 (0.003)	0.031 (0.003)	0.049 (0.003)	0.020 (0.003)	0.029 (0.003)
Fem	−0.197 (0.002)	−0.205 (0.002)	−0.206 (0.002)	−0.186 (0.002)	−0.181 (0.002)	−0.180 (0.002)
PUS*Fem	0.109 (0.004)	0.100 (0.005)	0.115 (0.005)	0.099 (0.004)	0.089 (0.004)	0.098 (0.004)
$\overline{{R }^{2}}$	0.43	0.42	0.41	0.52	0.49	0.48
No. obs	206,752	198,751	200,477	206,752	198,751	200,477

Estimation by pooled OLS. The set of controls in panel (a) includes age (3), region of residence (6), education (7) plus tenure and its square, while dummies for fixed-term contract (1), part-time work schedule (1), type of collective bargaining (3) and occupation (15) are also added in panel (b). Standard dev. in parentheses

We initially perform the OB decomposition for the whole sample of individuals in each wave of EES using the SR approach and some quantile regressions (Tables 6 and 7). Next, we run separate regressions distinguishing between individuals with at least a completed college degree (*college, engineers and PhDs* in Table 8) and those with lower educational attainments. Lastly, to examine whether the observed wage gap patterns by education vary across genders, the sample is also split by sex. In short, we compute the OB decomposition for the following four subsamples: men and women with college education, and men and women with lower educational attainments (Tables 9 and 10).

## Estimation results

First, Table 1 presents the pooled OLS estimates of the coefficients in the two specifications of the basic (hourly) wage regression in (1).

As can be inspected, the estimated coefficient on the public sector dummy, $PUS$, is positive and highly significant in all instances. Not surprisingly, the conditional public wage premium, which hovers between 2 and 5 logarithmic points (lp., hereafter), is much lower than the raw gaps shown in Fig. 1. The coefficient on the female dummy, *Fem*, implies that women suffer a wage penalty of about 18–21 lp*. vis-á-vis* men. Yet, the estimated coefficient on the interaction term, $Fem*PUS,$ is highly significant and positive (around 8–11 lp.), implying that working in the public sector almost halves the overall gender wage gap.

To analyse the potential role of omitted unobserved components (not captured by the observed controls) in biasing the estimates, we resort to Oster (2019)'s results on the relevance of this bias, based on coefficient movements scaled by the change in R-squared when extra controls are included in the regression (as in specification II). Assuming an equal selection relationship between observables and unobservables, and denoting the vector of estimated coefficients on$PUS$, $Fem$ and $PUS*Fem$ under specifications I and II by ${\widehat{{\varvec{\beta}}}}_{{\varvec{I}}}$ and${\widehat{{\varvec{\beta}}}}_{{\varvec{I}}{\varvec{I}}}$, respectively, this author derives a consistent estimator of ${\varvec{\beta}}$ given by the vector of adjusted estimates  with $\xi =\frac{{R}_{\mathrm{max}}^{2}-{R}_{II}^{2}}{{R}_{II}^{2}-{R}_{I}^{2}}$. In this expression ${R}_{I}^{2} ,$ ${R}_{II}^{2}$ and ${R}_{\mathrm{max}}^{2}$ are the R-squared from the two specifications and a hypothetical regression including all the relevant observables and unobservables, which Oster (2019) recommends setting equal to 1.3 ${R}_{II}^{2}$ in practice. For illustrative purposes, the estimates for 2010 yield  and ${\widehat{{\varvec{\beta}}}}_{{\varvec{I}}{\varvec{I}}}=\left(.049, -.186, .099\right)\boldsymbol{^{\prime}},$ which are fairly close. Similar results hold for the other two waves. Hence, even considering the potential endogeneity of some of the controls included in specification II, we take these findings as indicating that the role of unobservables in generating severe biases is minor. Consequently, for practical purposes, we keep the extended set of controls as the maintained specification in the sequel.

**Fig. 2:**
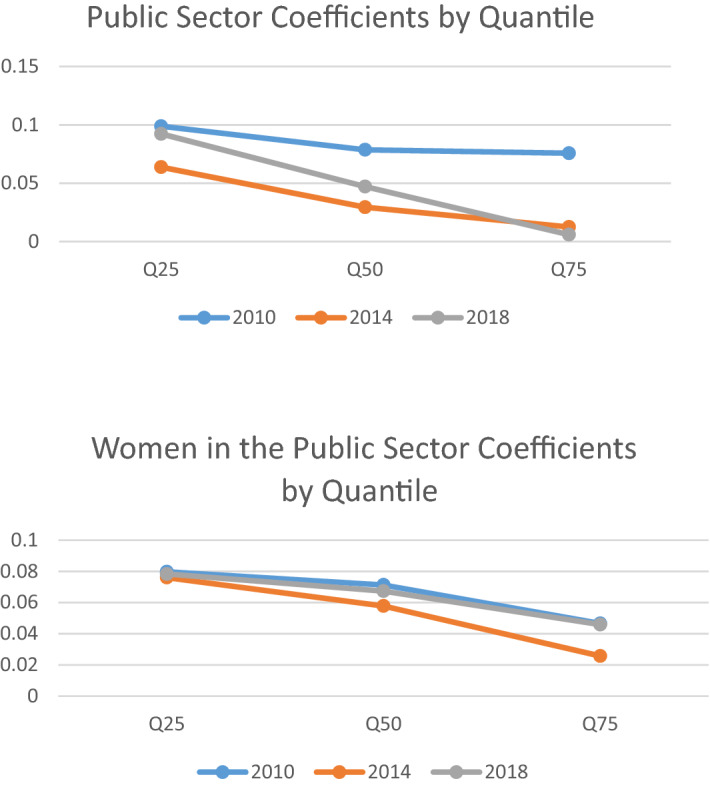
QR estimates of Public sector (upper panel) and Public sector* Female (lower panel) coefficients. Notes: QR estimates. The set of controls include dummies for age (3), gender (1), region of residence (6), education (7), fixed-term contract (1), part-time work schedule (1), type of collective bargaining (3) and occupation (15) plus tenure and its square

In addition to the previous average effects, Fig. 2 depicts quantile-regression (QR) point estimates (at the 25th, 50th and 75th quartiles) of the effects of public sector employment and gender on the conditional distribution of hourly wages, which are reported in Table 9 of the Appendix.

As can be inspected, both the coefficients on $Pub$ and $Pub*Fem$ are quite larger at the lower than at the other quartiles, suggesting a wage-compression effect that will be further explored in the next sections.

Second, Table 2 reports the OB decomposition by sector with selection correction (estimated by SR) for the whole sample of individuals.^7^ We distinguish between the coefficients effect (*unexplained*) and the difference in the SR selectivity terms (*selectivity*), where the marginal effects of the identifying variables in the selection equation are reported in Table 10 of the Appendix.

**Table 2:** Oaxaca-Blinder decomposition (whole sample)

	2010	2014	2018
Public sector	2.491 (0.002)	2.528 (0.002)	2.620 (0.002)
Private sector	2.318 (0.001)	2.307 (0.001)	2.358 (0.001)
Wage gap	0.173 (0.002)	0.221 (0.002)	0.262 (0.002)
Unexpl. gap	0.053 (0.004)	0.048 (0.004)	0.054 (0.003)
Selectivity	0.008 (0.005)	0.006 (0.007)	0.006 (0.005)
No. obs	206,752	198,751	200,477

SR estimation. The set of controls includes dummies for age (3), gender (1), region of residence (6), education (7), fixed-term contract (1), part-time work schedule (1), type of collective bargaining (3) and occupation (15) plus tenure and its square. The probit selection equation includes dummies for maternity and paternity leaves, age (7), education (7) and location (6). Standard dev. in parentheses

These estimates show that enjoying a maternity leave increases the probability of working in the public sector, while paternity leaves do not matter. Yet, one of the main findings in in this Table is that the selectivity term is not significant in either of the two wage equations, also having a rather negligible contribution to the OB decomposition in all the three waves.^8^ In general, the unexplained component accounts for 25–35% of the overall public–private wage gap. This figure is about half of the share reported by Hospido and Moral-Benito (2016, Table 3) for the MCVL that reaches an average of 60% during their sample period (2005–2012).

**Table 3:** Oaxaca-Blinder decomposition by education levels

	College			Non-college		
	2010	2014	2018	2010	2014	2018
Public sector	2.762 (0.003)	2.752 (0.003)	2.855 (0.005)	2.402 (0.003)	2.414 (0.003)	2.473 (0.002)
Private sector	2.650 (0.002)	2.661 (0.002)	2.732 (0.003)	2.195 (0.001)	2.225 (0.001)	2.262 (0.001)
Wage gap	0.112 (0.004)	0.091 (0.004)	0.123 (0.004)	0.207 (0.002)	0.189 (0.003)	0.211 (0.002)
Unexpl. gap	−0.021 (0.005)	−0.017 (0.005)	−0.013 (0.005)	0.126 (0.004)	0.087 (0.004)	0.102 (0.004)
Selectivity	−0.004 (0.009)	−0.003 (0.007)	0.004 (0.006)	0.006 (0.007)	0.003 (0.006)	0.005 (0.007)
No. obs	57,270	59,029	58,740	154,0322	139,722	141,737

SR estimation. The set of controls includes dummies for age (3), gender (1), region of residence (6), fixed-term contract (1), part-time work schedule (1), type of collective bargaining (3) and occupation (15) plus tenure and its square. The probit selection equation includes dummies for maternity and paternity leaves, age (7) and location (6)*.* Standard dev. in parenthesis

Third, we examine how the OB decomposition varies across educational levels using subsamples of college and non-college individuals. Table 3 reports both raw gaps and the corresponding OB decompositions with SR estimates, excluding education from the set of covariates. Anyhow, as in Table 2, the estimates on the selection terms are also not statistically significant. Regarding the raw gaps, those for less-educated individuals (20 lp.) almost double the high-educated ones' (11 lp.). Likewise, the unexplained part (around 10 lp.) points to higher returns in the public sector for the former, whereas, on the contrary, it is negative (−1.5 lp.) for the latter.

In agreement with the evidence shown in Fig. 2 (and in most of the literature), both of these findings confirm that the wage distribution in the public sector is compressed at different levels of education. In other words, in comparison with the private sector, the public sector pays relatively higher wages to workers with lower qualifications while it pays less to those with higher levels of education. In line with this result, another interesting finding is that one of the main factors contributing to the explained part of the raw public–private wage gaps is the type of wage-setting regulation prevailing among civil servants (*other forms* in Table 8) which covers 34–45% of public employees (against 2–4% in the private sector). In effect, this variable explains about 13% and 21% of that component for college and non-college individuals, respectively, while, e.g. differences in the incidence of temporary employment only contribute by −8% and −16%. Since union density in the public sector more than doubles that in the private sector (33% vs. 15%, see Vandaele 2019), we interpret this evidence as yielding some support to a *sword-of-justice* effect in the public sector where their stronger unions seek to reduce pay dispersion more than in the private sector (see Metcalf et al. 2001).

Finally, to analyse whether the public–private wage gap by education has a gender dimension, the sample is further split into men and women. Tables 4 and 5 report their SR estimates for those with lower and higher educational attainments, respectively. To do so, different selection equations are estimated for men and women using paternity and maternity leaves separately and excluding gender and education as regressors.

**Table 4:** Oaxaca-Blinder decomposition by gender (non-college workers)

	Females			Males		
	2010	2014	2018	2010	2014	2018
Public sector	2.296 (0.002)	2.310 (0.003)	2.386 (0.002)	2.481 (0.002)	2.496 (0.004)	2.549 (0.004)
Private sector	2.035 (0.004)	2.082 (0.005)	2.128 (0.004)	2.294 (0.003)	2.318 (0.006)	2.349 (0.005)
Wage gap	0.261 (0.003)	0.228 (0.003)	0.258 (0.003)	0.187 (0.004)	0.178 (0.004)	0.200 (0.004)
Unexpl. Diff	0.128 (0.007)	0.089 (0.007)	0.108 (0.007)	0.083 (0.005)	0.084 (0.006)	0.073 (0.006)
Selectivity	0.014 (0.005)	0.015 (0.006)	0.013 (0.006)	−0.004 (0.004)	0.003 (0.005)	0.004 (0.005)
No. Obs	64,652	61,597	62, 837	83,900	85,184	81,201

SR estimation. The set of controls includes dummies for age (3), region of residence (6), fixed-term contract (1), part-time work schedule (1), type of collective bargaining (3) and occupation (15). The probit selection equations includes dummies for maternity (women) and paternity (men) leaves, plus age (7) and location (6)*.* Standard dev. in parentheses

**Table 5:** Oaxaca-Blinder decomposition by gender (college workers)

	Females			Males		
	2010	2014	2018	2010	2014	2018
Public sector	2.705 (0.005)	2.713 (0.006)	2.805 (0.006)	2.865 (0.006)	2.856 (0.005)	2.926 (0.005)
Private sector	2.454 (0.007)	2.503 (0.006)	2.569 (0.008)	2.803 (0.007)	2.815 (0.006)	2.865 (0.006)
Wage gap	0.251 (0.005)	0.210 (0.006)	0.236 (0.005)	0.062 (0.005)	0.041 (0.006)	0.061 (0.006)
Unexp. diff	0.077 (0.008)	0.058 (0.007)	0.072 (0.007)	−0.026 (0.007)	−0.033 (0.006)	−0.042 (0.008)
Selectivity	0.007 (0.009)	0.010 (0.011)	0.013 (0.010)	−0.008 (0.007)	−0.003 (0.008)	−0.009 (0.008)
No. obs	25,343	24,146	24,629	29,108	27,824	31,830

SR estimation. The set of controls includes dummies for age (3), gender (1), nationality (1), region of residence (6), fixed-term contract (1), part-time work schedule (1), type of collective bargaining (3) and occupation (15) plus tenure and its square. The probit selection equation includes dummies for maternity and paternity leaves, age (7), location (6) and education (7)*.* Standard dev. in parentheses

Similar to the estimates in Table 3, the component capturing difference in returns among less-educated individuals (Table 4) is always positive yielding a wage premium of around 10 lp. for both men and women. However, when the focus is on high-educated individuals (Table 6), results differ markedly by gender. Women with a college degree enjoy an unexplained wage premium (7.0 lp.), while men suffer a wage penalty (−4.5 lp.). Moreover, a very interesting novelty in this decomposition is the large magnitude and high statistical significance of the difference in the selection terms for less-educated women. In particular, its positive sign implies that those unobserved female characteristics which increase the probability of public administration employment have a favourable impact on wages. On the contrary, that does not hold for high-educated women, possibly because their higher investment in human capital signals stronger commitment to the labour market. These findings partly agree with Hospido and Moral-Benito (2016)'s result that there was positive selection into the public sector at the bottom of the wage distribution during their sample period. However, we only find this evidence for less-skilled women and not for men. Moreover, unlike these authors, we do not observe negative selection at the top of the distribution. Finally, as regards temporary employment, its contribution to the explained part of the raw gap remains lower (in absolute value) than the contribution of the specific wage-settlement agreements in the public sector, even for less-educated women who are overrepresented in that type of employment (−13% vs. 17%).

**Table 6:** Public–private wage gap (whole sample and subsamples)

	2010	2014	2018
Whole sample	0.061	0.054	0.060
Whole sample (college)	−0.025	−0.020	−0.009
Whole sample (non-coll.)	0.132	0.090	0.107
Men (college)	−0.034	−0.056	−0.051
Women (college)	0.089	0.068	0.085
Men (non-college)	0.079	0.087	0.077
Women (non-coll.)	0.142	0.104	0.121

The reported wage gaps are the sum of the unexplained and selectivity components in the OB decompositions shown in Tables 3, 4 and 5

Taken together, these results support the presence of an unfavourable gender pay gap for less-educated women in the private sector. In this respect, Dolado et al. (2013) argue that, due to their lower job stability, women's lower wages could originate from receiving less training financed by firms or having to cover a larger share of the costs of their specific human capital. By contrast, the specific regulations in the public sector aimed at improving the balance of work and family lives turn this wage penalty into a wage premium.^9^

To wrap up all the previous evidence, Table 7 summarises the wage gaps for all the specifications considered above once differences in observed characteristics are accounted for. Overall, these results convey four main messages: (i) there remains a non-explained public wage premium of 6 pp. for the whole sample which is somewhat smaller in 2014, possibly due the wage reduction and freezing policies implemented between 2010 and 2015, (ii) this wage premium is enjoyed by workers with lower educational attainments while in contrast a small wage penalty is suffered by those with higher qualification, (iii) wage compression is a persistent phenomenon, and (iii) positive selection into the public sector mainly pertains to less-skilled women.

**Table 7:** OLS estimates of the mincerian wage regression with triple interaction (whole sample)

	Females			Males		
	2010	2014	2018	2010	2014	2018
Public sector	2.296 (0.002)	2.310 (0.003)	2.386 (0.002)	2.481 (0.002)	2.496 (0.004)	2.549 (0.004)
Private sector	2.035 (0.004)	2.082 (0.005)	2.128 (0.004)	2.294 (0.003)	2.318 (0.006)	2.349 (0.005)
Wage gap	0.261 (0.003)	0.228 (0.003)	0.258 (0.003)	0.187 (0.004)	0.178 (0.004)	0.200 (0.004)
Unexpl. diff	0.128 (0.007)	0.089 (0.007)	0.108 (0.007)	0.083 (0.005)	0.084 (0.006)	0.073 (0.006)
Selectivity	0.014 (0.005)	0.015 (0.006)	0.013 (0.006)	−0.004 (0.004)	0.003 (0.005)	0.004 (0.005)
No. obs	64,652	61,597	62, 837	83,900	85,184	81,201

OLS estimates of a mincerian (logged) hourly wage regressions for each of the three EES waves using a private sector and female dummies, double and triple interactions, plus the set of controls described in the note below Table 2. Standard dev. in parentheses

There are a few potential explanations consistent with this evidence. Some are based on different risk attitudes or other behavioural traits which we cannot control with our dataset (see Buurman et al. 2012). However, on the basis of the findings presented so far, we are able to suggest a few key ingredients which could help rationalise these results. First, the opposite sign of the wage gap by education can be explained by the public sector acting as a bilateral monopsonist in several economic activities, where it faces upward sloping labour supplies of its labour inputs. In this respect, a common finding in the literature is that the elasticity of labour supply for less-skilled workers is higher than for high-skilled workers, and that this difference increases the harder the skills and qualification required are (see Borjas 2002). Accordingly, monopsonistic power implies that, *vis-á-vis* a competitive equilibrium, the public sector reduces the wages of those workers with more inelastic labour supplies relative to those with higher elasticities. Second, the other leg of the bilateral monopsony is the presence of much stronger unions in the public than in the private sector, whose goal is to compress the wage distribution since their median voter is typically a public employee with a low or medium qualification levels. Finally, to explain the different results by gender, a plausible story is the one pointed out before. Women, particularly in their fecundity age, are subject to statistical discrimination in the private sector due to their greater job instability, while stricter regulations in the public sector prevent this type of actions.

To help think how these mechanisms can shape the results, a more formal analysis is sketched in the next section for illustrative purposes.

## Interpretation of empirical results

Let us consider a cost-minimising monopsonistic employer, akin to the public sector, with high ($H$) and less-skilled ($L$) workers subject to a Cobb–Douglas production function where parameter $\alpha >1$ captures the relative efficiency of $H$ workers, having normalised the efficiency of $L$ workers to 1.^10^ The employer faces inverse labour supplies denoted as ${w}_{H}\left(H\right)$ and ${w}_{L}\left(L\right)$, respectively. Hence, the demand of $H$ and $L$ workers solves the standard cost minimization problem:

2 $$ \min_{H,L} \left\{ {w_{L} \left( L \right)L + w_{H} \left( H \right)H} \right\} \,\,s.t.\,\,\, \overline{Y} = \left( {\alpha H} \right)^{\alpha } L^{1 - \alpha } , $$

whose f.o.c. are given by:

$$ w_{H} e_{H} = \lambda \alpha \frac{{\overline{Y}}}{H} \,\,\&\,\,\, w_{L} e_{L} = \lambda \left( {1 - \alpha } \right)\frac{{\overline{Y}}}{L} , $$

where $$\lambda $$ is the Lagrange multiplier and $$ e_{i}  = 1 + {\raise0.7ex\hbox{$1$} \!\mathord{\left/ {\vphantom {1 {d_{i} }}}\right.\kern-\nulldelimiterspace} \!\lower0.7ex\hbox{${\epsilon_{i} }$}},\,i{\mkern 1mu}  = {\mkern 1mu} H,L $$. Following the labour-supply arguments in Sect. 5, it is assumed that $${\epsilon }_{L}>{\epsilon }_{H},$$ so that $${e}_{H}>{ e}_{L}$$. Then,

3 $$ \frac{{w_{H} H}}{{w_{L} L}} = \frac{\alpha }{1 - \alpha } \frac{{e_{L} }}{{e_{H} { }}} < \frac{\alpha }{1 - \alpha } , $$

implying that the relative wage bill between $H$ and $L$ workers will be lower than in the competitive equilibrium, akin to the private sector, where ${e}_{L}={e}_{H}=1$ since both labour supplies are perfectly elastic, i.e.${\epsilon }_{L},{\epsilon }_{H}\to \infty .$

The corresponding labour demands (conditional on output) are as follows

4 $$ H = \left( {\frac{1 - \alpha }{\alpha }} \right)^{{ - \left( {1 - \alpha } \right)}} \left( {\frac{{w_{H} e_{H} }}{{w_{L} e_{L} }}} \right)^{{ - \left( {1 - \alpha } \right)}} \frac{{\overline{Y}}}{{a^{\alpha } }} , $$

5 $$ L = \left( {\frac{1 - \alpha }{\alpha }} \right)^{\alpha } \left( {\frac{{w_{H} e_{H} }}{{w_{L} e_{L} }}} \right)^{\alpha } \frac{{\overline{Y}}}{{a^{\alpha } }} . $$

Next, let us assume that wages of both types of workers are determined in a monopoly union model where a trade union maximises its utility function $\Omega$ subject to the above labour demand functions. As usual in this kind of wage-bargaining models, the union's goal is to maximise a combination of the wage surplus in relation to an alternative wage in the absence of agreement, $\overline{w }$ (which we take to be the competitive wage) and employment, with weights given by $\eta$ and $1-\eta$ for $H$ and $L$ workers, respectively. The novelty here that the utility function also includes the union's objective of achieving wage compression, captured by the quadratic penalty term ${0.5 \varphi (\mathrm{ln}{w}_{H}-ln{w}_{L})}^{2}$. Hence, the union solves the problem,

$$\max _{{W_{H} , W_{L} }} \Omega = [ \eta (
{\ln ( {w_{H} - \overline{w}_{H} } ) + \ln H} ) + ( {1 - \eta } )(
{\ln ( {w_{L} - \overline{w}_{L} } ) + \ln L} ) - \frac{\varphi
}{2}(\ln w_{H} - \ln w_{L} )^{2}]$$ subject to (4) and (5),whose corresponding f.o.c. are

$$ \frac{{\partial
{\Omega }}}{{\partial w_{H} }} = \left( {1 - \eta } \right)\alpha +
\eta \left[ \frac{{Z_{H} }}{{Z_{H} - 1}} - \left( {1 - \alpha } \right) \right]
- \varphi \ln (w_{H} /w_{L} ) = 0, $$

$$ \frac{{\partial {\Omega }}}{{\partial w_{L} }} = \eta \left( {1 - \alpha } \right) + \left( {1 - \eta } \right)\left[ {\frac{{z_{L} }}{{z_{L} - 1}} - \alpha } \right] + \varphi \ln (w_{H} /w_{L} ) = 0 $$

where $$z_{i} = w_{i} /\overline{w}_{i} .$$

Combining both f.o.c. yields, or alternatively,

$$ \frac{{z_{H} }}{{z_{L} }} = 1 + \frac{1}{{\varphi \ln (w_{H} /w_{L} ) - \alpha }} \equiv 1 + \delta (w_{H} /w_{L} ) $$

6 $$ \frac{{w_{H} }}{{w_{L} }} = \left( {1 + \delta \left( {w_{H} /w_{L} } \right)} \right)\frac{{\overline{w}_{H} }}{{\overline{w}_{L} }} $$

where $\delta \left(\cdot\right)$ is a decreasing function of the relative wage. Hence, differentiating both sides of (6) w.r.t. the wage-compression parameter $\varphi$ yields the required result: the relative wage of $H$ workers with respect to $L$ workers will be smaller than in the competitive equilibrium if $-1<\delta <0,$ which holds for a sufficiently high values of that parameter.

The previous model has not distinguished workers by gender. However, it is well documented in the literature (see, e.g. Alesina, et al. 2012) that male labour supply is much more inelastic (especially at the extensive margin) than female’s. Thus, the monopsonistic “exploitation” result in (3) on its own would apply to men more than women, which would go against an overwhelming evidence in favour of a positive male–female wage gap in most countries. Thus, an additional ingredient is needed to revert this result.

A plausible one is the statistical discrimination channel proposed by in De la Rica et al. (2007) and Dolado et al. (2013) that we summarise in what follows. Let us think of men and women being equally productive ex ante with productivity equal to 1, and that, to perform a job, those with less education require training, $\tau ,$ which is provided by the employer at a convex cost $c\left(\tau \right)={\tau }^{2}$ and increases productivity to 1 + $\tau$. The employer makes a wage offer, $w\left(\tau \right)$, which workers get before receiving a disutility shock, ω, which forces them to quit the job (say, for family duties) after being trained when $\omega \ge w\left(\tau \right).$ The $\omega$ shock is a random variable with c.d.f. $F(\omega$) which for simplicity is assumed to be uniform $U\left[0, \epsilon \right],$ with $\epsilon >$ 0.25 (see expression 8). The employer chooses the function $w\left(\tau \right)$ to maximise profits, $\Pi$, given by: 7 whose f.o.c. yields the optimal wage schedule ${w}^{*} \left(\tau \right)= \frac{1+\tau }{2}$. Then, replacing this expression into (7) and maximising again w.r.t.$\tau$ implies that the optimal training level and optimal wages are given by:

8 $$ \tau^{*}= \frac{1}{4 \epsilon -1 }\qquad \& \qquad w^{*}=\frac{1 - \tau^{*}}{2} $$

So that the higher ϵ, the lower ${\tau }^{*}$ and ${w}^{*}.$ Now suppose that employers in the private sector believe than women on average have a higher value of ϵ (i.e. a higher probability of quitting) than men. Then, to the extent that they observe imperfectly the individual ϵ′s they will offer a lower wage to this group of women (i.e. statistical discrimination) than in the public sector, where gender-equity regulations rule out that type of believes. Note that this type of argument would not apply to high-educated workers since they do not need training to perform the job.

To verify that statistical discrimination plays a key role in the private sector, we follow Altonji and Pierret (2001) who argue that such a type of discrimination should decrease as the individual is older or has longer job tenure. The insight is that employers should be able to learn much faster about the true productivity of more stable and senior workers because this learning investment process would be in their benefit. To do so, we run a similar regression to (1) but this time using a private sector dummy, $$PRI$$, its interaction with $$Fem$$ and its triple interaction with $$Fem$$ and tenure ($$Ten$$), $$PRI*Fem*Ten$$.

The results are gathered in Table 7 where we report the estimated coefficients in the model with the above-mentioned triple interaction. The coefficient on $Ten$ is 0.4 lp. Lower for females than for males in the private sector but the coefficients on the triple interaction term are all positive and statistically significant, suggesting that statistical discrimination exerted in the private sector seems a plausible hypothesis.

## Conclusions

This paper revisits and updates the existing evidence about the (hourly) wage gap between employees in the public and private sectors in Spain during and after the Great Recession. Using wage and demographics microdata from the three latest waves of the EES survey (2010, 2014, 2018), we compute standard and selectivity-corrected Oaxaca-Blinder decompositions in *mincerian* equations of hourly wages for each sector to estimate the wage gap component which is not explained by differences in observed productivity-related characteristics. In line with previous results for Spain and other southern European countries, we find a procyclical public wage premium due to differences in returns and endogenous selections of around six logarithmic points which has remained stable over the period under consideration. However, this premium is lower than the ones derived in other related studies using alternative data sources, as the MCVL. In agreement with previous findings, conclusive evidence is reported in favour of wage compression by education levels. When looking at gender, there is a public wage premium for females but only for non-college women. We interpret these results in terms of higher monopsonistic power of the public sector in regards men and high-educated workers (since they are likely to have less elastic labour supplies), greater unionisation and stronger preferences of less-skilled women to work in this sector in order to reconcile family and work.

## Appendix 1

See Tables 8, 9 and 10.

**Table 8:** Descriptive statistics of selected variables for the public and private sectors

Public sector	Distribution (%)		
	2010	2014	2018
*Occupations*			
A0. Directors and Managers	2.32%	2.28%	1.99%
B0. Health/Educ Tech. and Prof.	22.20%	24.01%	27.21%
C0. Other sci./int. Tech and Prof.	14.58%	14.76%	14.97%
D0. Technicians: Support Prof.	15.61%	15.85%	15.18%
E0. Office empl. not dealing with public	11.52%	11.42%	8.79%
F0. Customer services clerks	5.01%	3.27%	5.82%
G0. Catering/trade serv. workers	0.65%	0.54%	0.45%
H0. Health/Social Care workers	9.01%	8.12%	8.91%
I0. Prot. and security serv. workers	4.20%	5.11%	4.50%
J0. Skilled agricultural workers	0.52%	0.69%	0.65%
K0. Skilled construction workers	2.05%	1.73%	1.55%
L0. Skilled manuf. industry workers	2.14%	2.42%	1.82%
M0. Stationary plant/machine operators	0.31%	0.32%	0.18%
N0. Mob. machine drivers/operators	2.67%	2.96%	2.55%
O0. Unskilled service workers	5.51%	5.31%	3.99%
P0. Agricultural, fishing, const. labour	1.69%	1.20%	1.45%
*Gender*			
Male	46.15%	46.17%	43.79%
Female	53.85%	53.83%	56.21%
*Work schedule*			
Full-time	91.17%	90.58%	91.11%
Part-time	8.83%	9.42%	8.89%
*Type of contract*			
Permanent	66.88%	71.46%	66.64%
Fixed Term	33.12%	28.54%	33.36%
*Region*			
Northwest	11.13%	11.29%	10.08%
Northeast	11.08%	14.98%	14.16%
Madrid	14.86%	14.25%	13.57%
Central	15.89%	13.55%	16.22%
East	24.14%	24.60%	24.02%
South	17.66%	16.49%	17.18%
Canary islands	5.24%	4.84%	4.78%
*Education*			
None	1.25%	0.46%	0.25%
Primary	6.02%	4.51%	4.66%
Secondary I	17.06%	17.50%	15.82%
Secondary II	12.21%	15.19%	16.32%
Lower Vocational Training	8.45%	5.01%	3.00%
Upper Vocational Training	8.43%	8.56%	8.52%
Diploma	18.42%	18.72%	20.13%
College, Engineers and PhDs	28.16%	30.05%	31.30%
*Age*			
20–29	8.76%	5.19%	4.34%
30–39	29.36%	25.67%	18.17%
40–49	35.12%	34.88%	35.67%
50–59	26.76%	34.27%	41.82%
*Wage-Setting Regulation*			
Sectoral/State	10.25%	9.45%	9.01%
Sectoral at a lower level (regional, provincial,)	14.96%	15.05%	12.27%
Firm level	37.49%	35.16%	31.73%
Work centre level	3.69%	2.67%	4.27%
Other forms of regulation	33.61%	37.66%	42.72%
*Maternity/Paternity Leave Rate *			
Maternity	6.29%	6.50%	5.90%
Paternity	2.29%	2.80%	1.91%
Continuous variables	Mean (St. Dev.)		
Job Tenure (years)	11.03 (9.89)	13.30 (10.03)	14.65 (10.01)
Hourly wage (euros)	15.08 (8.53)	15.15 (8.16)	16.31 (8.51)
*Occupations*			
A0. Directors and Managers	3.57%	3.52%	3.42%
B0. Health/Educ Tech. and Profs.	2.65%	2.77%	2.56%
C0. Other sci./int. Tech and Profs.	9.56%	10.89%	10.35%
D0. Technicians: Support Professs.	18.83%	18.33%	16.42%
E0. Office empl. not dealing with public	8.58%	8.59%	6.90%
F0. Customer services clerks	4.50%	4.82%	7.01%
G0. Catering/trade serv. workers	7.59%	7.57%	8.00%
H0. Health/Social Care workers	4.70%	4.47%	5.07%
I0. Prot. and security serv. workers	1.90%	1.95%	1.82%
J0. Skilled agricultural workers	0.29%	0.27%	0.31%
K0. Skilled construction workers	3.76%	3.21%	3.55%
L0. Skilled manuf. industry workers	10.60%	10.20%	11.30%
M0. Stationary plant/machine operators	6.69%	7.97%	5.94%
N0. Mob. machine drivers/operators	4.67%	4.43%	3.97%
O0. Unskilled service workers	6.50%	6.27%	6.20%
P0. Agricultural, fishing, const. labour	5.61%	4.75%	7.18%
*Gender*			
Male	58.55%	58.77%	58.64%
Female	41.45%	41.23%	41.36%
*Work schedule*			
Full-time	83.21%	81.88%	81.80%
Part-time	16.79%	18.12%	18.20%
*Type of contract*			
Permanent	80.23%	81.39%	82.48%
Fixed Term	19.77%	18.61%	17.52%
*Region*			
Northwest	11.91%	11.58%	11.57%
Northeast	15.81%	15.73%	15.60%
Madrid	16.35%	16.48%	16.27%
Central	11.74%	11.51%	11.40%
East	27.02%	27.63%	27.76%
South	12.89%	12.83%	13.08%
Canary islands	4.29%	4.23%	4.26%
*Education*			
None	2.55%	1.40%	0.94%
Primary	13.65%	14.85%	16.04%
Secondary I	27.13%	24.34%	24.16%
Secondary II	12.27%	13.08%	14.40%
Lower Vocational Training	9.57%	10.0%	9.01%
Upper Vocational Training	10.35%	9.48%	10.57%
Diploma	9.41%	9.39%	9.83%
College, Engineers and PhDs	15.06%	17.46%	16.06%
*Age*			
20–29	18.33%	12.65%	11.60%
30–39	37.20%	35.28%	27.88%
40–49	27.47%	32.39%	36.22%
50–59	17.00%	19.68%	24.30%
*Wage-Setting Regulation*			
Sectoral State	30.03%	34.11%	36.35%
Sectoral at a lower level (regional, provincial, county.)	43.68%	39.58%	38.74%
Company level	19.85%	18.17%	18.31%
Work centre level	3.60%	4.13%	3.22%
Other forms of regulation	2.84%	4.01%	3.38%
Maternity/Paternity Leave Rate			
Maternity	5.49%	5.25%	3.69%
Paternity	4.16%	3.88%	2.32%
*Continuous variables*	Mean (St. Dev.)		
Job Tenure (years)	7.73 (8.66)	8.66 (8.59)	8.83 (8.83)
Hourly wage (euros)	13.06 (6.95)	12.64 (10.29)	13.22 (12.35)

**Table 9:** Quantile-regression estimates of the public–private sector wage gap for each of (whole sample)

EES 2010			
	Q25	Q50	Q75
PuS	0.0988 (0.003)	0.0786 (0.003)	0.0757 (0.004)
Fem	−0.1635 (0.002)	−0.1897 (0.002)	−0.2154 (0.002)
PuS*Fem	0.0797 (0.004)	0.0713 (0.004)	0.0466 (0.005)
*EES 2014*			
PuS	0.0638 (0.003)	0.0295 (0.003)	0.0126 (0.004)
Fem	−0.1493 (0.002)	−0.1696 (0.002)	−0.1955 (0.002)
PuS*FEM	0.0760 (0.004)	0.0578 (0.004)	0.0257 (0.005)
Fem	−0.1493 (0.002)	−0.1696 (0.002)	−0.1955 (0.002)
*EES 2018*			
Pus	0.0923 (0.003)	0.0472 (0.003)	0.0060 (0.004)
FEm	−0.1523 (0.002)	−0.1714 (0.002)	−0.1865 (0.002)
Pub*Fem	0.0783 (0.004)	0.0673 (0.004)	0.0459 (0.005)

Results of the estimation of a quantile regression of a mincerian (logged) hourly wage equation with controls shown in Table 2. Standard dev. in parentheses

**Table 10:** Probit average marginal effects (whole sample)

	2010	2014	2018
Maternity	0.039 (0.008)	0.032 (0.009)	0.041 (0.008)
Paternity	0.009 (0.009)	−0.006 (0.010)	0.007 (0.010)
Pseudo R2	0.223	0.191	0.237
No. obs	206,752	198,751	200,477

Probit estimation of selection into into the public sector ($\left(PUS=1\right)$. Additional controls are age (3), education (7) and location (6) dummies. Standard dev. in parentheses

## Footnotes

1 See Giordano et al. (2011) for an overview of these studies.

2 As discussed below, 2018 is the latest year in our dataset for which reliable microdata on public and private employees' hourly wages and demographics are available before the arrival of the Covid-19 pandemic which is left out of the analysis due to its own idiosyncratic nature.

3 In particular, working hours in the MCVL need to be imperfectly matched with data on hours drawn from the *Spanish Labour Force Survey* (Encuesta de Población Activa– EPA), possibly leading to measurement errors in the computation of hourly wages.

4 Note that the share of temporary employment has been traditionally higher in the private sector, except at the beginning of the Great Recession when a massive destruction of temporary jobs hit private firms. Subsequently, fixed-term contracts grew in the public sector (especially though interim hiring in Education and Health) to supposedly undertake provisional and specific needs, particularly by town halls and provincial councils.

5 Note that choice of occupations could be arguably endogenous. Nonetheless, they could also be considered predetermined variables and, as an accounting exercise, it is useful to know the extent to which the wage gap can be explained by them since they also affect productivity.

6 The reference categories in the estimation are: age (50–59), gender (male), nationality (foreign), education (college, engineers and PhDs), region (Madrid), contract (open ended), work schedule (full time), collective bargaining (state) and occupation (directors and managers).

7 The first two rows in Table 2 report average (logged) hourly wages in each of the EES waves. Therefore, the reported wage gap in lp. is the difference between the figures presented in those two rows.

8 The Wald test confirms the null hypothesis of random selection into the public sector with p-values above 0.30.

9 As mentioned earlier, Gomes (2015) argues that this finding clashes with the theory of compensating differentials in a competitive framework which would predict lower wages in the public sector, unless better working regulations improve productivity thorough, e.g. an efficiency wage channel.

10 It can be easily shown that the same qualitative results hold when a CES production function is assumed.
